# Immuno-protective impact of Kangfuxin liquid on systematic toxicity associated with autologous hematopoietic stem cell transplantation in multiple myeloma

**DOI:** 10.3389/fmed.2025.1700829

**Published:** 2025-12-17

**Authors:** Xianyi Wu, Mingxuan Tang, Taohua Deng, Fujun Qu, Fang Jiang, Qin Zhou, Wenyuan Lin, Xiaotao Wang

**Affiliations:** 1 Department of Hematology, The Affiliated Hospital of Guilin Medical University, Guilin, China; 2 Graduate College of Guilin Medical University, Guilin, China; 3 Department of Pain Therapy, The Affiliated Hospital of Guilin Medical University, Guilin, China

**Keywords:** autologous hematopoietic stem cell transplantation, high-dose melphalan, Kangfuxin liquid, multiple myeloma, toxicity

## Abstract

**Objective:**

This study evaluated the effectiveness of Kangfuxin liquid against autologous stem cell transplantation toxicity in patients with multiple myeloma.

**Methods:**

This single-center retrospective study involved 82 participants, divided into the simple oral cryotherapy group (control group), which included 43 patients with multiple myeloma who underwent autologous hematopoietic stem cell transplantation before May 2023, and the Kangfuxin liquid + oral cryotherapy group (experimental group), which included 39 patients with multiple myeloma, who underwent autologous hematopoietic stem cell transplantation after June 2023. Statistical analysis indicators included infection, gastrointestinal toxic side effects, efficacy evaluation, and prognostic analysis.

**Results:**

Compared with the control group, the experimental group had a higher mean age (*p* = 0.003), fewer reinfused stem cells (*p* < 0.001), and a longer neutrophil and platelet engraftment time (*p* = 0.001 and p < 0.001). The experimental group had lower auxiliary treatment costs (*p* < 0.0001), and significantly lower infection incidence (*p* = 0.001) and mucositis and diarrhea grading (*p* = 0.046), indicating a protective effect. There were no additional adverse reactions, and the treatment efficacy for the patients’ primary diseases remained unaffected.

**Conclusion:**

Kangfuxin liquid significantly reduces mucosal damage, shortens hospitalization, lowers medical costs, and does not compromise the therapeutic efficacy of autologous transplantation for multiple myeloma, providing evidence for clinical treatment.

## 1 Introduction

Multiple myeloma (MM) is one of the most common hematologic malignancies. Although MM prognosis has improved significantly in recent years with the development and application of drugs like proteasome inhibitors, immunomodulators, and bispecific antibodies (1), it remains incurable. Studies have shown that, as a consolidation therapy, autologous hematopoietic stem cell transplantation (auto-HCT) can enhance remission on the basis of novel drug treatments and improve the therapeutic efficacy of MM (2). It remains one of the most effective methods for delaying disease relapse (3).

The high-dose melphalan (HDM) conditioning regimen is the preferred protocol for patients with MM before auto-HCT. This is because of its high efficacy, low relapse rate, prolonged survival, and minimal exacerbation of myelosuppression (4, 5). However, HDM and auto-HCT are associated with mucositis symptoms, including oral ulcers, vomiting, and diarrhea, which are their primary dose-limiting toxicities (6). Severe toxic reactions prolong hospitalization, increase treatment costs, exacerbate healthcare burdens, reduce patient satisfaction, and can adversely affect treatment outcomes (7, 8). Therefore, drugs or methods that improve mucosal toxicity in these patients are necessary.

Kangfuxin liquid, a traditional Chinese medicine preparation from *Periplaneta americana*, is primarily used to treat digestive system ulcer-related diseases, including gastric ulcers and duodenal ulcers (9). Additionally, reports (10, 11) demonstrate its notable protective effects against mucosal damage by radiotherapy and chemotherapy. Here, we report the protective role of Kangfuxin liquid in mitigating HDM therapy-induced mucosal injury. Our results indicate that it significantly reduces mucosal damage, shortens hospitalization duration, and lowers medical costs without compromising the therapeutic efficacy of autologous transplantation for MM. This finding provides stronger evidence to support clinical diagnosis and treatment.

## 2 Patients and methods

### 2.1 Study population

This study retrospectively analyzed 82 patients with MM who underwent auto-HCT with the HDM conditioning regimen at the Department of Hematology, Affiliated Hospital of Gui’lin University, from October 1, 2020, to March 31, 2025. Consecutive admissions were included to minimize selection bias. All patients met the MM diagnostic criteria established by the International Myeloma Working Group (12). Risk stratification was not performed because some patients did not undergo Fluorescence *In Situ* Hybridization testing for economic reasons.

The inclusion criteria were as follows: (a) age 18–75 years, (b) confirmed MM diagnosis, (c) receipt of HDM as conditioning chemotherapy before auto-HCT, and (d) availability of complete clinical and laboratory data. The exclusion criteria included: (a) incomplete medical records, (b) loss to follow-up before engraftment, (c) coexistence of other hematologic malignancies or severe cachexia, and (d) deviation from the HDM conditioning protocol. This study was approved by the Medical Ethics Committee of the Affiliated Hospital of Guilin Medical University (No 2024KJTLL-33), and it strictly adhered to the principles of the Declaration of Helsinki. All patients gave informed consent (via telephone or by signing written informed consent forms), agreeing to have their data used for scientific research.

### 2.2 Treatment

*Standard treatment regimen*: All patients underwent HDM as preconditioning chemotherapy. The dose was calculated based on renal function, and the total amount administered was maintained between 140 mg/m^2^ and 200 mg/m^2^. Other medications, such as prophylactic antiemetics and organ-protective drugs, were routinely administered, and most drug names and doses were consistent across both groups.

The control group included patients who underwent autologous HCT between January 2022 and May 2023, whereas the KFX group included patients treated between June 2023 and March 2024. During this period, institutional protocols for conditioning regimen (high-dose melphalan), infection prophylaxis, transfusion thresholds, and nursing care remained unchanged. No policy modifications related to antibiotic prophylaxis, mucositis grading, or hospitalization procedures occurred during the study interval.

KFX (production unit: Sichuan Good Doctor Panxi Pharmaceutical Co., Ltd.; National Medicine Standard Z51021834; composition: extract of *Periplaneta americana*; solvent: sterile water) was supplied in 100 mL single-use bottles. The solution was stored at 4 °C and thawed to room temperature before use.

The KFX group patients were instructed to use 10 mL as an oral rinse for approximately 15 s immediately before melphalan infusion and every 6 h thereafter for 5 consecutive days. The solution was swished for 15 s and then swallowed. Administration was supervised by trained nursing staff, and adherence was confirmed by bedside nurses and medical record review.

All patients across groups received consistent standard supportive care. This included antiemetics (ondansetron 8 mg I q24h or granisetron 1 mg IV q24h), antibiotic prophylaxis (levofloxacin 500 mg daily for high-risk neutropenia), antifungal prophylaxis (fluconazole 400 mg daily), and analgesics (acetaminophen or opioids, as needed for mucositis). All patients used standard oral rinses (saline or bicarbonate-based), and ice-saline cryotherapy was identical in both groups. Palifermin was not administered in any patient. Transfusion thresholds were standardized as a platelet level of <20 × 10^9^/L with hemoglobin <70 g/L.

### 2.3 Evaluation criteria

The evaluation criteria for treatment-related adverse reactions were assessed according to the National Cancer Institute’s Common Terminology Criteria for Adverse Events version 5.0 (13). A body temperature of >37.3 °C (measured in the armpit) was defined as fever, and the site of infection was assessed based on symptoms. Infections were evaluated and classified according to the CDC/NHSN surveillance definitions and IDSA guidelines for febrile neutropenia in hematologic malignancies. Bloodstream infection (BSI): Defined as at least one positive blood culture for a recognized pathogen accompanied by compatible clinical manifestations (e.g., fever ≥ 38 °C, chills, or hypotension). Intestinal infection: Defined as ≥3 episodes of watery or loose stools per day for ≥2 consecutive days, with or without an identified enteric pathogen, after excluding chemotherapy-induced mucositis or drug-related diarrhea. Pulmonary infection: Defined as new or progressive pulmonary infiltrates on imaging, together with fever, cough, or dyspnea, with or without microbiological confirmation from sputum or bronchoalveolar lavage cultures. The duration of each adverse reaction was recorded. The number of days from the start of preconditioning to discharge was considered the period of hospitalization for hematopoietic stem cell transplantation. The costs incurred because of infections or other complications were calculated as supportive care expenses. Supportive care costs were obtained from the hospital information system and included expenses related to medications (antiemetics, antibiotics, growth factors), blood product transfusions, laboratory and imaging tests, and hospitalization costs (including ICU stay when applicable). Costs were expressed in Chinese Yuan (CNY) and standardized to 2023 values using the national medical CPI. Non-medical costs were excluded. For reference, costs were also converted to US dollars (USD) using an exchange rate of 1 USD = 7.1 CNY. Neutrophil and platelet engraftment times were evaluated by monitoring complete blood count results. A sustained absolute neutrophil count of at least 0.5 × 10^9^/L for 3 days was considered to indicate neutrophil engraftment. A sustained count of at least 20 × 10^9^/L platelets for 7 days without platelet transfusion was considered to indicate platelet engraftment. MM treatment efficacy was assessed using the International Myeloma Working Group response criteria, including complete response, very good partial response, partial response, stable disease, and progressive disease (14). Progression-free survival (PFS) after transplantation was defined as the time from diagnosis to disease progression or death from any cause. Overall survival (OS) was defined as the time from diagnosis to the last follow-up or death from any cause.

### 2.4 Follow-up

The patients were monitored continuously and followed up until April 30, 2025. PFS and OS were determined using follow-up data collected by reviewing inpatient and outpatient medical files, and through phone follow-ups. The median follow-up after transplantation was a (a-a) months.

### 2.5 Statistical analyses

Statistical analyses were performed using SPSS version 27.0 (IBM Corp., Armonk, NY, United States) and GraphPad Prism version 10 (GraphPad Software, San Diego, CA, United States). Categorical variables were presented as frequencies and percentages, and comparisons between groups were made using the chi-square test or Fisher’s exact test, as appropriate. Normally distributed continuous variables were expressed as mean ± standard deviation (SD) and compared using the independent-samples *t*-test, whereas non-normally distributed data were expressed as median (interquartile range) and compared using the Mann–Whitney *U*-test. Multivariable logistic regression analyses were used to evaluate factors associated with grade 3–4 mucositis and infection, and multivariable linear regression was applied to assess factors influencing hospitalization cost. Covariates included treatment group, age, and CD34^+^ cell dose. Results are presented as adjusted odds ratios (ORs) or regression coefficients (B) with 95% confidence intervals (CIs). Engraftment outcomes were analyzed using the Kaplan–Meier method and compared by the log-rank test. A two-sided *p*-value < 0.05 was considered statistically significant.

Post-hoc power analysis: Because this was a retrospective study, no *a priori* sample size estimation was performed. Post-hoc power analysis was conducted using G*Power version 3.1 based on the observed effect sizes of the primary endpoints (severe oral mucositis and infection) and the secondary endpoint (supportive care cost). The analysis used a two-tailed *α* = 0.05 and the actual sample sizes of both groups (*n* = 39 and *n* = 43).

The median follow-up time was calculated using the reverse Kaplan–Meier method. Progression-free survival (PFS) was defined as the time from transplantation to disease progression or death from any cause, whichever occurred first. Overall survival (OS) was defined as the time from transplantation to death from any cause. Patients alive and progression-free at the last follow-up were censored at that date. Survival probabilities were estimated using the Kaplan–Meier method, and group comparisons were performed using the log-rank test. The number of patients at risk was displayed below each survival curve.

## 3 Results

### 3.1 Clinical features

This retrospective cohort study enrolled 82 patients (median age: 57 years, range: 38–71 years), and of these, 35 (42.7%) were male and 47 (57.3%) were female. Based on immunoglobulin (Ig) subtype, 48 (58.5%), 17 (20.7%), 2 (2.4%), 11 (13.4%), and 4 (4.9%) cases were IgG, IgA, IgD, light chain, and non-secretory, respectively. Disease staging according to the Durie–Salmon criteria revealed that 7 (8.5%), 34 (41.5%), and 41 (50.0%) patients were in stage I, II, and III, respectively. Classification based on the International Staging System showed that 8 (9.8%), 29 (35.4%), and 45 (54.9%) patients were in stage I, II, and III, respectively. R-International Staging System was not applied because of incomplete genetic testing data in early-stage patients. All patients received frontline induction chemotherapy, and those who achieved at least a partial response proceeded to auto-SCT. A comparison of the clinical characteristics of the two patient groups revealed that only age was significantly different (*p* = 0.003). Table 1 summarizes patient characteristics, including diagnostic criteria, staging, treatment regimens, and pre-transplant efficacy assessments.

**Table 1:** The baseline clinical characteristics of the enrolled patients.

Characteristics	Control group (*n* = 43)	Experimental group (*n* = 39)	Test	*p*
Gender				
Male	19 (44.2%)	16 (41%)	*χ*^2^ = 0.083	0.826
Female	24 (55.8%)	23 (59%)		
Mean age (mean ± SD)	55.28 ± 6.77	59.87 ± 6.96	T = −3.028	0.003
M protein type			*χ*^2^ = 0.289	0.991
IgG	26 (60.5%)	22 (56.4%)		
IgA	9 (20.9%)	8 (20.5%)		
IgD	1 (2.3%)	1 (2.6%)		
Light chain	5 (11.6%)	6 (15.4%)		
Non-secretory	2 (4.7%)	2 (5.1%)		
DS stage			*χ*^2^ = 0.102	0.950
I	3 (7.0%)	4 (10.3%)		
II	19 (44.2%)	15 (38.5%)		
III	21 (48.8%)	20 (51.2%)		
ISS stage			*χ*^2^ = 0.138	0.933
I	4 (9.3%)	4 (10.3%)		
II	16(37.2%)	13 (33.3%)		
III	23 (53.5%)	22 (56.4%)		
Efficacy before HCT			*χ*^2^ = 3.999	0.135
CR	2	7		
VGPR	27	23		
PR	14	9		

### 3.2 Hematopoietic reconstitution

Hematopoietic reconstitution assessment: all patients completed myeloablative conditioning chemotherapy with HDM and subsequently underwent stem cell infusion. Comparative analysis revealed that the number of CD34 + cells infused (*p* < 0.000) and the time to neutrophil and platelet engraftment (*p* = 0.001 and <0.000, respectively) were significantly different in the control vs. experimental groups. However, the infused mononuclear cell count (*p* = 0.844) was not significantly different between the two groups. Table 2 details the statistical data comparing the two groups.

**Table 2:** The number of reinfused stem cells and the cell engraftment time.

Group	CD34 + count (×10^6^/kg)	MNC count (×10^8^/kg)	neutrophil engraftment (days)	Platelet engraftment (days)
Control (*n* = 43)	6.78 ± 3.42	4.95 ± 3.03	10.02 ± 1.46	11.40 ± 1.95
Experimental (*n* = 39)	4.74 ± 1.67	4.82 ± 3.36	11.05 ± 1.12	13.67 ± 2.68
Text	3.371	0.197	−3.554	−4.415
*p*	<0.000	0.844	0.001	<0.000

In multivariable logistic regression analysis, Kangfuxin (KFX) treatment remained an independent protective factor against both severe mucositis and post-transplant infections. Specifically, KFX significantly reduced the odds of grade 3–4 mucositis (OR = 0.118, 95% CI 0.029–0.488, *p* = 0.003) (Table 3) and infection (Table 4) (OR = 0.115, 95% CI 0.030–0.439, *p* = 0.002) after adjusting for age, CD34^+^ dose, disease stage, and other clinical variables. Among covariates, higher ISS stage was independently associated with increased infection risk (OR = 9.476, 95% CI 1.319–68.070, *p* = 0.025), while other variables showed no significant associations.

**Table 3:** Multivariate logistic regression analysis of factors associated with severe oral mucositis (grade ≥3) after autologous hematopoietic stem cell transplantation.

Variable	B	S.E.	Wald	Sig.	Exp(B)	95% CI for Exp(B)
Group (KFX vs control)	−2.137	0.724	8.706	0.003	0.118	0.029–0.488
Age	−0.077	0.047	2.637	0.104	0.926	0.844–1.016
CD34 dose	0.035	0.099	0.126	0.723	1.036	0.853–1.257
Gender	−1.002	0.613	2.669	0.102	0.367	0.11–1.221
DS	1.3	0.916	2.016	0.156	3.671	0.61–22.093
ISS	−1.294	0.917	1.992	0.158	0.274	0.045–1.653
Pre-transplant therapeutic effect	−0.671	0.522	1.652	0.199	0.511	0.184–1.422

**Table 4:** Multivariate logistic regression analysis of factors associated with infection after autologous hematopoietic stem cell transplantation.

Variable	B	S.E.	Wald	Sig.	Exp(B)	95% CI for Exp(B)
Group (KFX vs. control)	−2.167	0.686	9.978	0.002	0.115	0.03–0.439
Age	−0.017	0.042	0.166	0.684	0.983	0.906–1.067
CD34 dose	0.078	0.117	0.446	0.504	1.081	0.86–1.359
Gender	−0.152	0.567	0.071	0.789	0.859	0.283–2.612
DS	−1.871	0.985	3.611	0.057	0.154	0.022–1.061
ISS	2.249	1.006	4.997	0.025	9.476	1.319–68.07

### 3.3 Safety assessment

All enrolled patients successfully achieved hematopoietic reconstitution, and there was no transplantation-related mortality. HDM preconditioning chemotherapy was associated with common adverse reactions, including hematologic and non-hematologic toxicities. Regarding hematologic toxicities, all patients developed grade 4 neutropenia and thrombocytopenia. There were 3 (7.7%) and 5 (11.6%) cases of Grade 3 anemia in the experimental and control group, respectively. There were no Grade 4 anemia cases in either group. There were 75 (91.5%) cases with neutropenia and fever, of which 17 (20.7%) had bloodstream infections and 40 (48.8%) had intestinal infections. The comparison between the two groups showed statistically significant differences (*p* < 0.001). All infections were effectively controlled regardless of site and grade, and there were no infection-related deaths. Non-hematological adverse reactions predominantly involved the digestive system, with 82 (100%) and 72 (87.8%) cases experiencing nausea and vomiting, respectively, without significant differences between the two groups. A total of 79 cases (96.3%) suffered from oral mucositis, and comparison between the two groups showed statistically significant differences (*p* < 0.001). Additionally, 82 cases (100%) had diarrhea, and the comparison between the two groups showed statistically significant differences (*p* = 0.046). There were no adverse reactions related to the heart, liver, kidneys, or coagulation system. Table 5 shows the statistical grading of adverse reactions in the two groups.

**Table 5:** Comparison of safety between the two groups of patients during auto-HCT.

Adverse event	Control group (*n* = 43)	Experimental group (*n* = 39)	Test	*p*
Infection			39.083	<0.001
Blood	12	5		
Intestinal	25	15		
Vomiting			6.322	0.097
0	3	7		
I	5	8		
II	23	20		
III	12	4		
IV	0	0		
Oral mucositis			19.684	<0.001
0	0	3		
I	5	18		
II	20	13		
III	15	5		
IV	3	0		
Diarrhea			7.981	0.046
0	0	0		
I	4	7		
II	18	24		
III	19	8		
IV	2	0		

### 3.4 Comparison of supportive treatment expenses

Upon comparing the supportive treatment costs between the two groups of patients, statistical data were calculated in Renminbi and analyzed in yuan. The results indicated that the supportive treatment costs were higher in the control group than in the experimental group. Further comparison revealed that supportive treatment costs were significantly different between the two groups (*p* = 0.000). Table 6 shows a detailed comparison of supportive treatment costs.

**Table 6:** Comparison of supportive treatment expenses.

Group	Supportive care chemotherapy	95%CI
Control (*n* = 43)	28,231 ± 5,780	26999.37–28288.6
Experimental (*n* = 39)	22,502 ± 8,243	21443.1–23884.74
*t*	3.671	–
*p*	0.000	–

In multivariate linear regression analysis, Kangfuxin (KFX) treatment was independently associated with a significant reduction in total hospitalization cost after auto-HCT (B = −6530.184, *p* < 0.001). After adjusting for age, CD34^+^ cell dose, and ISS stage, the KFX group showed an average cost reduction of approximately 6,500 CNY compared with the control group. Other covariates, including age, CD34^+^ dose, and ISS stage, were not significantly associated with total cost (*p* > 0.05) (Table 7).

**Table 7:** Multivariate linear regression analysis of factors associated with cost after autologous hematopoietic stem cell transplantation.

Variable	B	S.E.	*t*	Sig.
Group (KFX vs. control)	−6530.184	1789.779	−3.649	<0.001
Age	51.362	116.858	0.440	0.662
CD34 dose	−276.214	295.978	−0.933	0.354
ISS	−804.342	1193.651	−0.674	0.502

### 3.5 Comparison of therapeutic efficacy before and after transplantation

The clinical efficacy of the two groups of patients was evaluated before auto-HCT and 3 months post-transplantation. Evaluation timing could be adjusted for some patients based on actual follow-up visits and the follow-up cutoff time. Therapeutic efficacy improved in both groups before and after transplantation, although they did not differ significantly. Table 8 shows the clinical efficacy data of the 82 patients before and after auto-HCT.

**Table 8:** Comparison of therapeutic efficacy before and after transplantation.

Group	sCR/CR		VGPR		PR	
	Before HCT	After HCT	Before HCT	After HCT	Before HCT	After HCT
Control	2	7	19	27	22	9
Experimental	5	10	20	26	14	3
Text	0.336		0.045		0.759	
*p*	0.562		0.833		0.384	

### 3.6 Post-hoc power analysis

Post-hoc power calculations showed that the study achieved a power of 0.94 for detecting the difference in severe oral mucositis incidence (41.9% vs. 83.7%) and 0.92 for infection incidence (51.3% vs. 88.4%). For supportive treatment costs (22,502 ± 8,243 vs. 28,231 ± 5,780 yuan), the effect size (Cohen’s *d* = 0.79) yielded a power of 0.87. These results indicate that the sample size was adequate to identify significant and clinically relevant differences between the two groups.

### 3.7 Prognosis analysis

**Figure 1:**
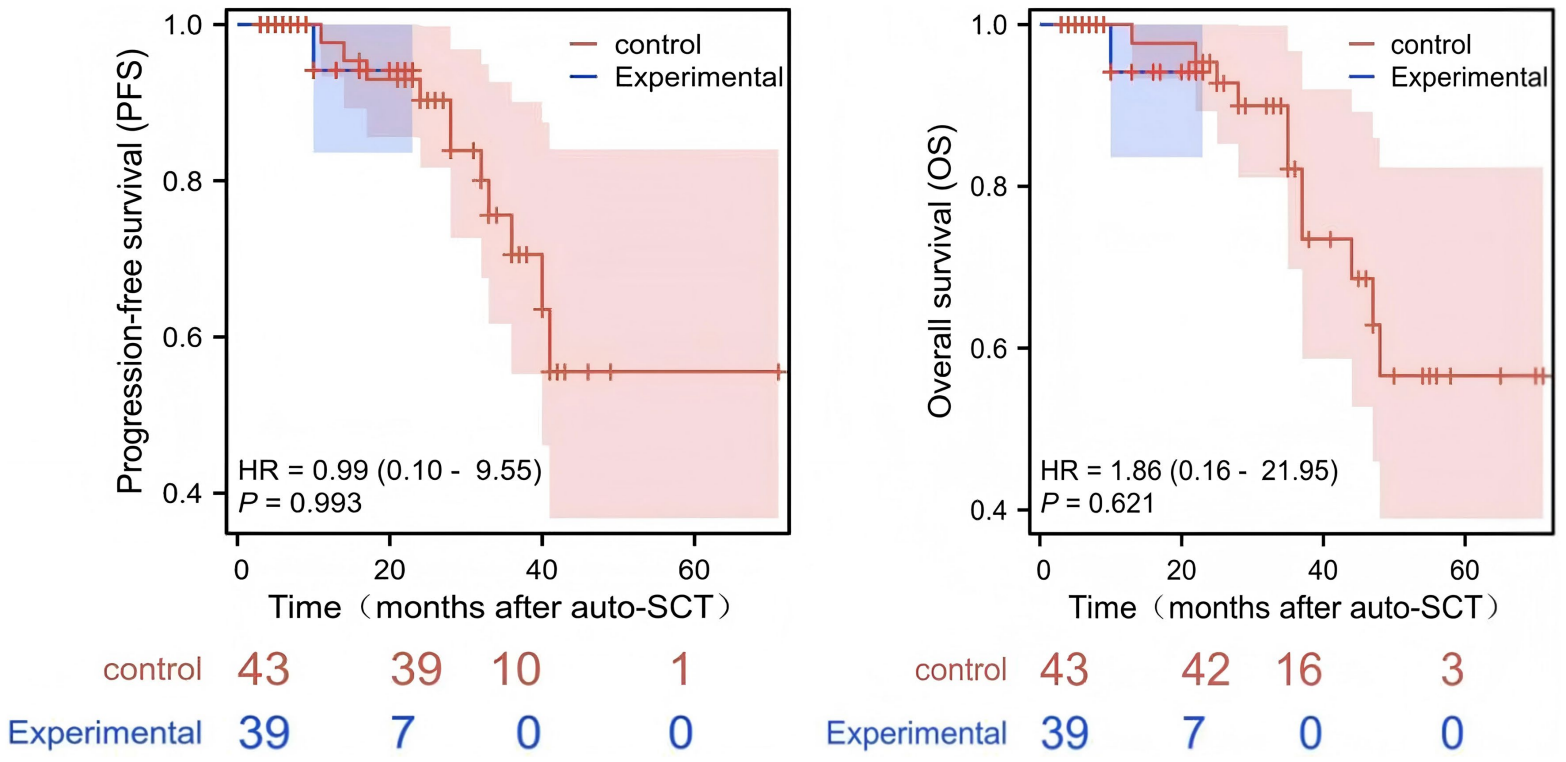
Survival analysis of the two patient groups after auto-HCT.

All patients were continuously monitored until May 31, 2025. Follow-up data were collected by reviewing inpatient and outpatient medical records, as well as via telephone interviews, to evaluate efficacy and analyze PFS and OS. At the time of data cutoff (May 31, 2025), the median follow-up duration was 26.5 months (95% CI: 23.4–29.6 months). PFS and OS were not significantly different between the two groups (*p* = 0.417 and 0.157, respectively; Figure 1).

## 4 Discussion

MM is a common plasma cell disorder that accounts for approximately 10% of all malignant hematological diseases (15). Despite significant advancements in MM survival rates thanks to novel drug therapies, auto-HCT retains a central role. Moreover, for patients with MM undergoing transplantation, HDM remains the standard conditioning regimen.

The efficacy and toxicity profile of the HDM conditioning regimen have garnered widespread academic attention. Because HDM conditioning is myeloablative, patients with MM inevitably experience myelosuppression upon receiving HDM conditioning. Neutropenia increases the infection risk, while thrombocytopenia raises the risk of bleeding. Hematologic toxicity is an unavoidable side effect (6). Mounting research is focused on HDM’s non-hematologic toxicities, which commonly manifest as oral mucositis, vomiting, diarrhea, etc. If not effectively prevented and controlled, non-hematologic toxicities can severely compromise patient quality of life, increase medical costs, and may even become life-threatening in severe cases. Therefore, minimizing HDM’s non-hematologic toxicities is a shared pursuit among all hematopoietic stem cell transplantation practitioners.

This study compared patients with MM who underwent auto-HCT after HDM conditioning, with or without Kangfuxin liquid, to determine differences in toxic side effects and therapeutic efficacy. The aim was to explore whether Kangfuxin liquid reduces the toxic side effects of HDM conditioning during auto-HCT while ensuring therapeutic efficacy.

A summary analysis of the clinical characteristics of the two groups revealed that the patients’ average age was significantly higher in the experimental group than in the control group. This phenomenon benefits from the recent relaxation of age restrictions for auto-HCT in patients with MM (16, 17). Older patients with MM can also benefit from auto-HCT if they are assessed as fit (16–18). The primary consideration is that the average age of MM onset is high (65 years) and auto-HCT is an important treatment modality for this disease (1, 2, 19). In recent years, the age limit for auto-HCT in patients with MM at our center has been gradually relaxed to benefit more older patients, and the current maximum age is 71 years.

Infusing an adequate quantity of stem cells is crucial to ensure successful cell engraftment and hematopoietic stem cell transplantation (20). Our study validated that the number of infused stem cells correlates significantly with the time to neutrophil and platelet engraftment. Additional research indicates that infusing sufficient stem cells is closely associated with patient prognosis, and a minimum hematopoietic stem cell count of 2.5 × 10^6^/kg is recommended for reinfusion (21). In this study, stem cell quantities exceeded this threshold in both groups. Further statistical analysis revealed that although the experimental group received significantly fewer stem cells than the control group, therapeutic efficacy and prognosis did not differ between the two groups. This phenomenon is linked to our center’s comprehensive preparation (since 2023) for tandem transplantation in high-risk patients with MM or second transplants for relapsed cases. Current studies indicate that high-risk patients with MM benefit from tandem transplantation, and patients with relapse after autologous transplantation benefit from a second transplant, provided sufficient stem cells are collected before the first transplant for both procedures (22–24). Therefore, our center ensures ample stem cell reserves for tandem or second transplants without compromising the initial transplantation’s efficacy, which explains the observed disparity in reinfused stem cell quantities between the two groups.

Gastrointestinal toxicities, such as diarrhea, nausea, and vomiting, are the most common non-hematological adverse reactions to melphalan, which impact nearly all patients to some degree (25, 26). Severe gastrointestinal toxicity can damage mucosal integrity, resulting in intestinal dysbiosis and an elevated risk of enteric infections (27). Moreover, bacterial translocation to the bloodstream may lead to bacteremia (28, 29). These issues degrade the patient’s transplant experience, lengthen the hospitalization period, and escalate supportive care expenses (30). Cryotherapy is the main strategy for gastrointestinal toxicity mitigation. However, the occurrence of Grade 2 toxicities (or higher) substantially restricts the pool of eligible patients and the selection of optimal drug dosages (31). This study found that in the experimental group, cryotherapy combined with the application of Kangfuxin solution could significantly reduce the incidence and severity of oral mucositis and diarrhea, greatly alleviating gastrointestinal toxic reactions. Kangfuxin liquid is rich in various active components, including epidermal growth factors, amino acids, nucleotides, and antimicrobial peptides (9). This formulation effectively accelerates the repair and healing of damaged tissues and wounds by fostering cell and blood vessel regeneration, cell proliferation, and new granulation tissue growth (9). Studies indicate that Kangfuxin liquid enhances immune function, reduces radiation damage, alleviates pain, and exerts significant anti-inflammatory effects (10, 32, 33). The protective effect of Kangfuxin solution on mucous membranes and its reparative function on mucositis have been effectively confirmed in our report, which is consistent with relevant research findings. Kangfuxin liquid has significant protective and reparative effects on mucous membranes (34, 35). Its cellular mechanism may involve reducing the levels of proinflammatory factors, e.g., TNF-α, IL-6, and IL-1β, thereby decreasing inflammation in the mucosal tissues and protecting and repairing the mucous membranes (36, 37). More importantly, our analysis revealed a statistically significant difference in infection rates between the two groups of patients. Bloodstream, pulmonary, and intestinal infection incidence was significantly lower in the experimental group vs. the control group. This is attributable to Kangfuxin solution’s excellent protective effects on the oral and gastrointestinal mucosa, which reduces mucositis and diarrhea occurrence, thereby indirectly decreasing the incidence of digestive tract dysbiosis and bacterial translocation. Studies have indicated that Kangfuxin liquid possesses both anti-infection and anti-inflammatory effects (37, 38). Kangfuxin liquid can inhibit microbial growth and development while promoting IL-17A release and neutrophil counts, thereby exerting its anti-infection effects (37). Moreover, the raw material of Kangfuxin liquid, *Periplaneta americana*, contains periplanetasin-5, an antimicrobial peptide (39) that can effectively control inflammation by inhibiting the phosphorylation of MAPK, an inflammatory signaling factor, and reducing IκB degradation (39). These factors may also explain the lower infection incidence in the experimental group. The supportive treatment costs were significantly lower in the Kangfuxin liquid group because it had lower gastrointestinal toxicity and infection incidence when compared with the control group, which alleviated the patients’ financial burden to some extent. These advantages in the Kangfuxin liquid group are established on the premise of not compromising treatment efficacy, which is reflected in our analysis of therapeutic outcomes and prognosis.

Beyond its mucosal protective effects, Kangfuxin liquid may also exert immunomodulatory activity that could be relevant to tumor immunity and immune cell interactions. Previous studies have demonstrated that Kangfuxin and its bioactive components from *Periplaneta americana* can regulate macrophage polarization (40), improve immune imbalance, and modulate cytokine production, including TNF-α, IL-6, and IL-10 (36, 41, 42). These processes are closely associated with immune surveillance and cellular communication phenomena such as trogocytosis, which contribute to antigen presentation and cytotoxic responses in the tumor microenvironment (43, 44). Although our study did not directly assess immune parameters, it is plausible that Kangfuxin’s ability to maintain mucosal immune balance and suppress excessive inflammation may also indirectly influence immune-mediated mechanisms during autologous transplantation. Future mechanistic studies are warranted to clarify the relationship between Kangfuxin and immune regulatory pathways involved in cancer immunity.

Based on our analysis, the use of HDM conditioning combined with Kangfuxin liquid during auto-HCT in patients with MM is safe and effective. While ensuring no treatment efficacy and prognosis compromise, this regimen significantly reduces gastrointestinal toxicities of Grade II and higher, effectively lowers infection risks, and decreases additional hospitalization costs. This improves patient quality of life and may encourage more patients with MM to opt for auto-HCT, ultimately enhancing survival outcomes.

This study has several limitations. First, it was a retrospective, single-center analysis with a relatively small sample size, which may limit the generalizability of the findings. Second, because control patients were treated before May 2023 and KFX-treated patients after June 2023, potential temporal bias cannot be completely excluded. Changes in clinical practice, infection control measures, or supportive care protocols over time may have influenced patient outcomes. Third, the baseline imbalance in CD34^+^ cell dose between groups may have partially contributed to differences in engraftment kinetics. Fourth, the study was not blinded, and the follow-up duration was relatively short, which limits the ability to assess long-term survival or relapse outcomes.

To validate and extend these observations, we plan to conduct a prospective, multicenter clinical study with larger cohorts and longer follow-up. We anticipate that in patients with relapsed or high-risk multiple myeloma undergoing melphalan-based autologous hematopoietic stem cell transplantation (auto-HCT), concomitant Kangfuxin liquid administration may safely allow melphalan dose escalation, thereby enabling more patients to benefit from intensified conditioning.

Although this study focused on clinical outcomes, the biological mechanisms by which Kangfuxin liquid mitigates mucosal and hematopoietic toxicity remain to be elucidated. In future work, we plan to perform *in vitro* assays using human oral epithelial cells to assess the effects of KFX on cell proliferation, migration, and wound healing under melphalan exposure. In addition, cytokine profiling of epithelial and immune cells treated with KFX will be conducted to explore its potential immunomodulatory and anti-inflammatory actions. These studies will help clarify the molecular pathways involved and further support the translational application of KFX in stem cell transplantation.

## Data Availability

The original contributions presented in the study are included in the article/supplementary material, further inquiries can be directed to the corresponding author.
