# Supplementary material for: Immuno-protective impact of Kangfuxin liquid on systematic toxicity associated with autologous hematopoietic stem cell transplantation in multiple myeloma
Source: Front Med (Lausanne). 2025 Dec 17;12:1700829. doi: 10.3389/fmed.2025.1700829 (PMC12753403; doi:10.3389/fmed.2025.1700829)
Supplement: Supplementary file 1 [file Data_Sheet_1.docx]

Supplementary Material

# Supplementary Data

Without

# Supplementary Figures and Tables

## Supplementary Tables

Table S1. Comparison of adverse events between groups with effect sizes and 95% Cis

| Adverse Event | Control (n=43) | Experimental (n=39) | Absolute Risk (%) | ARR (95% CI) | RR (95% CI) | OR (95% CI) | P value |
| --- | --- | --- | --- | --- | --- | --- | --- |
| Infection | 39 (90.7%) | 20 (51.3%) | 39.4% | 39.4% (22.8-56.0) | 0.57 (0.41-0.79) | 0.11 (0.03-0.37) | <0.001 |
| Oral mucositis (grade II–IV) | 38 (88.4%) | 18 (46.2%) | 42.2% | 42.2% (26.0-58.4) | 0.52 (0.35-0.75) | 0.13 (0.04-0.40) | <0.001 |
| Diarrhea (grade II–IV) | 39 (90.7%) | 32 (82.1%) | 8.6% | 8.6% (−5.0-22.2) | 0.90 (0.76-1.07) | 0.43 (0.09-1.91) | 0.046 |

Table S2. Comparison of supportive care cost between groups with effect sizes and 95% Cis

| Variable | Control (n=43) | Experimental (n=39) | Mean Difference (95% CI) | Cohen’s d | t | p value |
| --- | --- | --- | --- | --- | --- | --- |
| Supportive care cost (¥) | 28,231 ± 5,780 | 22,502 ± 8,243 | −5,729 (−8,709 to −2,749) | 0.81 | 3.67 | 0.0001 |

Table S3. Sensitivity Analyses of Clinical Outcomes After Autologous Hematopoietic Stem Cell Transplantation

| Analysis | Subset / Adjustment Method | Outcome | Adjusted OR (95% CI) | *p* Value |
| --- | --- | --- | --- | --- |
| (a) CD34⁺ ≥ 4.0 × 10⁶/kg | Restricted to adequate graft dose | Grade 3–4 oral mucositis | 0.24 (0.07–0.75) | 0.015 |
|  |  | Infection incidence | 0.28 (0.09–0.83) | 0.022 |
| (b) Temporal restriction | Excluded earliest and latest 10% of cases | Grade 3–4 oral mucositis | 0.26 (0.09–0.79) | 0.017 |
|  |  | Infection incidence | 0.33 (0.11–0.92) | 0.035 |
| (c) Propensity-score weighting | Weighted for age, CD34⁺ dose, and diagnosis date | Grade 3–4 oral mucositis | 0.27 (0.09–0.77) | 0.015 |
|  |  | Infection incidence | 0.31 (0.11–0.85) | 0.023 |

TableS4. Subgroup Analyses of Clinical Outcomes and Cost in Control vs Experimental.

| **Subgroup** | **Group** | **n** | **Survival=1** | **Infection=1** | **Oral mucositis ≥3** | **Cost (median, USD)** | **P values (G1 vs G2)** |
| --- | --- | --- | --- | --- | --- | --- | --- |
| **Age <60** | Control | 31 | 7 | 26 | 12 | 27,726 | Survival: 0.229  **Infection: 0.020**  **Oral mucositis: 0.017**  **Cost: 0.008** |
|  | Experimental | 18 | 1 | 9 | 1 | 21,845 | - |
| **Age ≥60** | Control | 12 | 4 | 11 | 6 | 27,166 | **Survival: 0.047**  **Infection: 0.027**  Oral mucositis: 0.114  Cost: 0.059 |
|  | Experimental | 21 | 1 | 11 | 4 | 19,570 | - |
| **CD34 Q1** | Control | 6 | 1 | 5 | 3 | 23,184 | - |
|  | Experimental | 15 | 0 | 7 | 2 | 21,835 | Survival: 0.286  Infection: 0.178  Oral mucositis: 0.115  Cost: 0.302 |
| **CD34 Q2** | Control | 10 | 2 | 9 | 4 | 31,959 | - |
|  | Experimental | 10 | 2 | 5 | 0 | 19,389 | Survival: 1.000  Infection: 0.141  Oral mucositis: 0.087  **Cost: 0.014** |
| **CD34 Q3** | Control | 12 | 5 | 12 | 7 | 30,099 | - |
|  | Experimental | 8 | 0 | 4 | 0 | 17,112 | Survival: 0.055  **Infection: 0.014**  **Oral mucositis: 0.015**  **Cost: 0.003** |
| **CD34 Q4** | Control | 15 | 3 | 11 | 4 | 26,241 | - |
|  | Experimental | 6 | 0 | 4 | 3 | 31,972 | Survival: 0.526  Infection: 1.000  Oral mucositis: 0.354  Cost: 0.569 |
| **ISS 1** | Control | 4 | 1 | 3 | 0 | 25,296 | - |
|  | Experimental | 4 | 0 | 3 | 0 | 21,562 | Survival: 1.000  Infection: 1.000  Oral mucositis: 1.000  Cost: 0.343 |
| **ISS 2** | Control | 15 | 3 | 13 | 7 | 27,562 | - |
|  | Experimental | 13 | 0 | 9 | 3 | 23,671 | Survival: 0.226  Infection: 0.372  Oral mucositis: 0.254  Cost: 0.214 |
| **ISS 3** | Control | 24 | 7 | 21 | 11 | 28,392 | - |
|  | Experimental | 22 | 2 | 8 | 2 | 18,952 | Survival: 0.139  **Infection: 0.001**  **Oral mucositis: 0.008**  **Cost: 0.002** |

## Supplementary Figures

Figure 1 Abstract figure


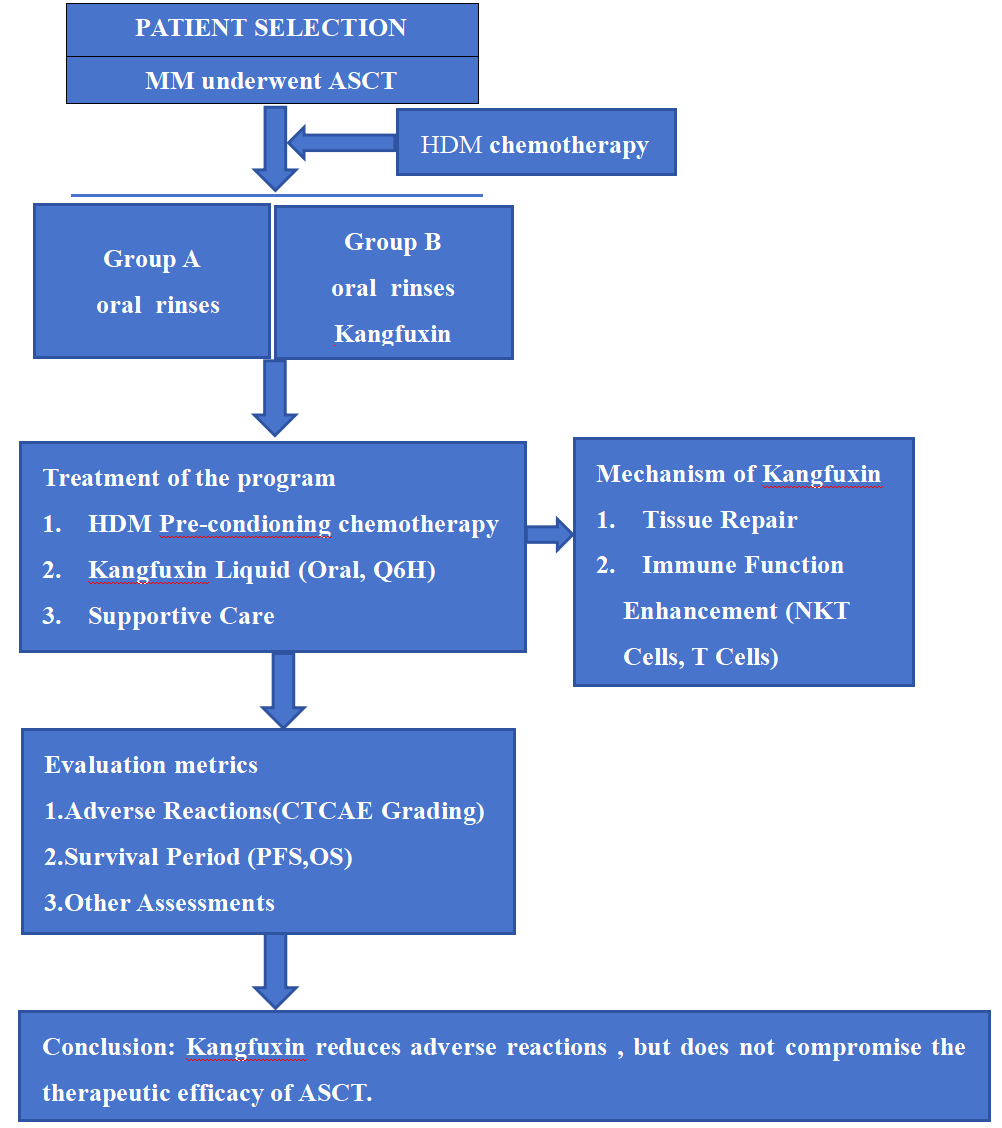


**
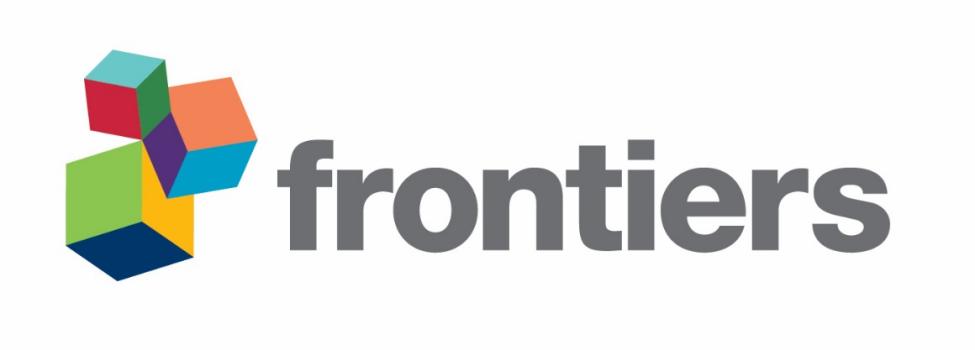
**

**Supplementary Figure 1.** The figure legends are required to have the same font as the main text, 12 point normal Times New Roman, single spaced. Please use a single paragraph for each legend and prepare the figures keeping in mind the PDF layout.
